# Effect of prior Zika and dengue virus exposure on the severity of a subsequent dengue infection in adults

**DOI:** 10.1038/s41598-022-22231-y

**Published:** 2022-10-14

**Authors:** Braulio M. Valencia, Ponsuge C. Sigera, Praveen Weeratunga, Nicodemus Tedla, Deepika Fernando, Senaka Rajapakse, Andrew R. Lloyd, Chaturaka Rodrigo

**Affiliations:** 1 School of Biomedical Sciences/Kirby Institute, UNSW Sydney, Sydney, NSW 2052 Australia; 2 Faculty of Medicine, University of Colombo, Colombo, CO008 Sri Lanka

**Keywords:** Dengue virus, Clinical microbiology

## Abstract

Given the structural similarity between Zika and dengue viruses, prior infection from one virus is hypothesized to modulate the severity of a subsequent infection from the other virus. A previous paediatric cohort study observed that a prior Zika infection may increase the risk of a subsequent symptomatic or severe dengue infection. The Colombo Dengue study is a prospective hospital-based cohort study in Sri Lanka that recruits symptomatic adult dengue patients within the first three days of fever. Anti-Dengue Envelope and anti-Zika NS1 IgG antibodies were tested by ELISA (Euroimmun, Lubeck, Germany) in all recruited patients. Associations between pre-morbid seroprevalence for either or both infections and adverse clinical outcomes of the current dengue infection were explored. A total of 507 dengue infected patients were assessed of whom 342 (68%) and 132 (26%) patients had anti-dengue IgG and anti-Zika IgG respectively. People with combined prior dengue and zika exposure as well as prior dengue exposure alone, were at increased risk of plasma leakage, compensated and uncompensated shock, and severe dengue (p < 0·05), compared to people without prior exposure to either infection. The effect of prior Zika exposure alone could not be established due to the small the number of primary dengue infections with prior Zika exposure.

## Introduction

Dengue and Zika viruses (DENV and ZIKV) are structurally similar flaviviruses^1^. There is evidence that prior infection from one virus may influence the severity of a subsequent infection from the other^2–4^. In DENV infection where there are four serotypes, infection with one serotype induces long-lasting immunity against that serotype but not others. Thus, a second infection with a heterologous serotype may lead to more severe disease due to cross reactive, non-neutralising antibodies enhancing pathogenicity via antibody dependent enhancement^5^. There is emerging evidence that prior ZIKV infection may also induce cross-reactive non-neutralising antibodies to influence the severity of a subsequent DENV infection, like a different DENV serotype^6^.

Clinical evidence for supporting this hypothesis comes from a single, but adequately powered paediatric cohort in Nicaragua which showed that children with previous ZIKV infection had a 12.1% greater probability of a subsequent symptomatic DENV infection as opposed to a 9.2% probability in children with a previous DENV infection (vs. 3.5% probability in children not exposed either infection)^6^. However, all dengue infections (n = 293) in this cohort which backed the above observation were sampled from a single epidemic in 2019/20 and were of DENV serotype 2 only.

For the reverse sequence of infection (i.e. DENV followed by ZIKV), in vitro and some animal studies suggest that prior DENV infection enhances the risk of severity of a subsequent ZIKV infection^2–4^. However, evidence from non-human primate models^7^ or from human studies^8–10^ suggest that a DENV-ZIKV infection sequence does not lead to more symptomatic or severe ZIKV infection. In fact, epidemiological evidence suggests that previous dengue infection may be protective against subsequent symptomatic ZIKV infection^11^. This protection is likely to be mediated via cross-reactive CD8 + T cells^12^. Both structural and non-structural DENV epitopes are recognised by T cell receptors and the latter is more likely to induce cross-reactivity after natural infection or vaccination due to the conserved nature of these epitopes^13^. ZIKV reactive T cells increase quickly and to a greater magnitude during acute ZIKV infection if the individual is also immune to DENV (compared to DENV naïve patients)^3^.

The Colombo Dengue Study (CDS) is a single centre prospective cohort study in Sri Lanka recruiting acute symptomatic adult dengue patients within the first 3 days of fever. The present study aimed to explore the prevalence of anti-ZIKV NS1 IgG and anti-DENV envelope IgG antibodies in these patients, to test the hypotheses if prior ZIKV infection alone, or the combination of a prior ZIKV and DENV infection would increase the severity of a subsequent DENV infection.

## Methods

### Study design and setting

**Figure 1:**
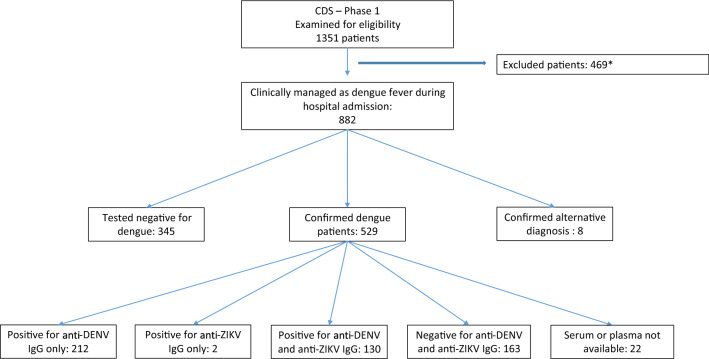
Flow chart showing patient recruitment to CDS and the numbers that tested positive for anti-DENV and ZIKV IgG. *Patients not meeting inclusion criteria of CDS (e.g., not presenting within the first three days of fever).

Clinical samples for this seroprevalence study were sourced from the Colombo Dengue Study (CDS), which is a collaborative project between University of Colombo, Sri Lanka and University of New South Wales, (UNSW) Sydney, Australia. This single centre prospective cohort study recruited a total of 523 virologically confirmed DENV infections (predominantly serotypes 1, 2 and 3) between 2017 and 2020 from the National Hospital of Sri Lanka (in Colombo, Sri Lanka). Only patients who were clinically suspected of having dengue fever and presented within the first three days of fever were enrolled. Patients were followed up daily until discharge to record the changes in clinical parameters and laboratory test results. The primary outcome for this cohort study was the occurrence of dengue associated plasma leakage, and the secondary outcomes were occurrence of severe dengue^14^, compensated or uncompensated shock, and death. Plasma leakage was defined as greater than 20% increase in haematocrit during hospital stay, or an absolute haematocrit count ≥ 45% at any time, or detection of pleural/peritoneal fluid collections by ultrasonography. Compensated shock was defined as pulse pressure equal to or less than 20 mmHg while a systolic blood pressure < 90 mmHg (or a decline by more than 20%) defined uncompensated shock. Dengue infection was confirmed by NS1 antigen testing (SD Bioline dengue NS 1 antigen test, Alere SD, USA) and RT-PCR^15^ for all patients. RT-PCR was done retrospectively (after the acute illness) and therefore all patients were later categorised in to two groups as dengue fever (DF—positive by either NS1 test or RT-PCR) and non-dengue fever (NDF—negative by both tests) (Fig. 1). This process replicates the real-world management of dengue patients in Sri Lanka since RT-PCR is not available as a clinical diagnostic test. Details of CDS including the rationale for sample size calculation and inclusion/exclusion criteria for patient recruitment had been published previously^16^. Previous investigations with data and samples from this cohort have been helpful to design cheaper diagnostics for dengue fever^16^, to design a new pan-serotype assay for dengue full genome sequencing^17^, characterise the syndrome of post-dengue fatigue^18^, to develop a triaging system to screen patients with ultrasonography in resource limited settings for plasma leakage^19^ and to model the direct costs of managing dengue patients in Sri Lanka^20^. All methods were performed in accordance with the relevant guidelines and regulations.

### Serology tests for prior DENV and ZIKV infections

EDTA plasma samples from all confirmed DF patients (1 per patient) were tested for anti-ZIKV NS1 IgG antibodies and anti-envelope DENV IgG antibodies using Euroimmun (Lubeck, Germany) anti-Zika IgG ELISA (catalogue number: EI 2668-9601) and anti-dengue type 1–4 IgG ELISA (catalogue number: EI 266a-9601-1) kits, according to manufacturer’s instructions^21,22^. Both qualitative and quantitative readouts were obtained. Aliquots of each sample were tested with both kits. All samples were collected from symptomatic patients during the first three days of fever, which is an inadequate time window for anti-dengue IgG to form due to the current infection^23,24^. Therefore, any positive results were attributed to a previous infection. The DENV IgG assay does not differentiate between antibodies against different DENV serotypes. NDF patients were not tested.

### Data analysis

As published previously, CDS maintains a comprehensive database of clinical (signs, symptoms, laboratory test results, outcomes), demographic and virological (infecting serotype and viral load) data linked to each patient in REDCap (Version 9·1, Vanderbilt University, USA) in a server hosted by the University of Colombo, Sri Lanka^20^. Descriptive statistics were summarized as mean (or median) and standard deviation (or interquartile range). Statistical associations were explored with the chi square test across two groups for dichotomous outcomes. Comparison of continuous outcomes between two groups was done with independent T tests or Wilcoxon signed-rank test depending on the normality of distribution of data arrays. Relative risk of adverse outcomes was calculated between the group with prior exposure to both infections vs. those with prior exposure to DENV only or those without prior exposure to both infections. Statistical significance was set at p < 0·05 and calculated as Pearson chi square two-tailed p value unless specified otherwise in text. If the number of observations in a cell of the 2 × 2 table was < 5, Fishers exact test was applied to calculate the p value. Finally, logistic regression was used to observe the collective influence of infecting dengue serotype, age, gender and prior DENV and ZIKV exposure (independent variables) on adverse clinical outcomes (dependent variables) during the current DENV infection. All analyses were performed with IBM SPSS Statistics (Version 25, USA).

### Ethics declarations

Ethics approvals for the study was obtained from the Ethics Review Committee, Faculty of Medicine, University of Colombo (EC/17/080), the Ethics Review Committee, National Hospital of Sri Lanka (ETH/COM/2017/12), and the Human Research Advisory Panel (Biomedical) of the University of New South Wales (HC180015, HC190513). All patients had given written informed consent to the donation and storage of serum samples.

## Results

A total of 529 confirmed dengue infections (males: 359, 67·9%, mean age: 31·3 ± SD: 13·6 years) were identified during Phase 1 of CDS (Fig. 1). Samples were not available from 4·2% (22/529) of patients; so, the remainder (n = 507) were tested for both anti-ZIKV and anti-DENV IgG. The primary outcome, plasma leakage was identified in 58·3% (260/446, missing: 83) of patients. Similarly, 4·7% (21/446) and 2·1% (11/446) of patients had compensated and uncompensated shock respectively, while 5·8% (26/446) met the criteria for severe dengue^14^. There were no deaths. The infecting serotype was characterised by RT-PCR in 426 individuals, and a majority (98·9%, 420/426) were infected by one serotype while six samples had two serotypes simultaneously detected. Majority of infections were of DENV serotype 2 (260/426, 61%), followed by serotype 3 (109/426, 25·6%) and serotype 1 (50/426, 11·7%). The characteristics of recruited patients are shown in Table 1.

**Table 1:** Characteristics of patients.

Characteristics	Number	Percentage (%)	Mean/median (SD or IQR)^a^
Age (in years)			31.3 (1.36)
**Sex**			
Male	359	67.9	
Female	170	32.1	
**Self-reported episodes of prior dengue (n-454)**			
Yes	27	5.9	
No	427	94.1	
**Dengue serotype of current infection (n-426)**			
DENV1	50	11.7	
DENV2	260	61	
DENV3	109	25.6	
Serotype unassigned	12	2.8	
Previous dengue infection (anti-dengue IgG + , n-507)	342	67.5	
Median anti-dengue IgG titre of positive cases (RU/ml)			179.6 (128.1–227.5)
Previous Zika virus infection (anti-Zika IgG + , n-507)	132	26	
Median anti-Zika IgG titre of positive cases (RU/ml)			83.1 (47.9–120.8)
**Adverse outcomes of current dengue infection (n-446)**			
Plasma Leakage	260	58.3	
Compensated shock	21	4.7	
Uncompensated shock	11	2.1	
Severe dengue	26	5.8	

^a^*SD* standard deviation, *IQR* inter-quartile range.

Plasma from 67·5% (342/507) of the patients were positive for anti-DENV IgG, although only 5·9% (27/454) of patients self-reported a prior dengue infection. Plasma was positive for anti-Zika IgG in 26% (132/507) of patients, and most of these (130/132, 98·5%) were also positive for anti-DENV IgG. The quantitative anti-DENV IgG reading was significantly higher in those exposed to both infections previously, compared to those only exposed to DENV (median: 218·3 vs. 165·9 RU/ml, p < 0·001 calculated with Wilcoxon signed-rank test). Patients with anti-DENV IgG were statistically significantly older than those without these antibodies (32·7 vs. 28·5 years, p = 0·001). However, a similar difference was not seen between patients with and without anti-ZIKV IgG (32·3 vs. 30·9 years, p = 0·321).

The effect of prior ZIKV exposure alone on the severity of a primary DENV infection could not be tested due to the inadequate number of eligible patients (n = 2). Prior DENV infection alone was associated with an increased risk of plasma leakage (RR: 1.73, 95% CI 1.38–2.17, p < 0.0001), compensated shock (p = 0.001), uncompensated shock (p = 0.04) and severe dengue (p < 0.001). A relative risk could not be calculated for latter three outcomes as the number of primary dengue patients having these adverse outcomes were zero. Having both prior ZIKV and DENV exposure was also associated with an increased risk of plasma leakage (RR: 1.46, 95% CI 1.13–1.9, p < 0.0001), compensated shock (p = 0.019), uncompensated shock (p = 0.042) and severe dengue (p = 0.002). The risk of adverse outcomes was similar between the group with dual exposure and the group with prior DENV exposure only (Tables 2, 3, 4 and 5).

**Table 2:** Association between previous DENV / ZIKV exposure and plasma leakage during the current DENV infection.

Anti-DENV/ZIKV IgG sero-status	Plasma leakage		Risk ratio (95% CI)	P value*
	No	Yes		
Prior DENV and ZIKV exposure	45	66	1.46 (1.13–1.89)	0.003
No exposure to both infections	79	54		
Prior DENV exposure only	53	126	1.73 (1.38–2.17)	< 0.001
No exposure to both infections	79	54		
Prior DENV and ZIKV exposure	45	66	0.84 (0.71–1.01)	0.073
Prior DENV exposure only	53	126		

*Calculated as Pearson chi square 2-tailed p value.

**Table 3:** Association between previous DENV / ZIKV exposure and uncompensated shock during the current DENV infection.

Anti-DENV/ZIKV IgG sero-status	Uncompensated shock		Risk ratio (95% CI)	P value*
	No	Yes		
Prior DENV and ZIKV exposure	107	4	Not calculated	0.042
No exposure to both infections	133	0		
Prior DENV exposure only	173	6	Not calculated	0.04
No exposure to both infections	133	0		
Prior DENV and ZIKV exposure	107	4	1.08 (0.29–3.91)	1.000
Prior DENV exposure only	173	6		

*Calculated as two-tailed p value with Fisher’s exact test.

**Table 4:** Association between previous DENV/ZIKV exposure and compensated shock during the current DENV infection.

Anti-DENV/ZIKV IgG sero-status	Compensated shock		Risk ratio (95% CI)	P value*
	No	Yes		
Prior DENV and ZIKV exposure	106	5	Not calculated	0.019
No exposure to both infections	133	0		
Prior DENV exposure only	166	13	Not calculated	0.001
No exposure to both infections	133	0		
Prior DENV and ZIKV exposure	106	5	0.62 (0.23–1.69)	0.455
Prior DENV exposure only	166	13		

*Calculated as two-tailed p value with Fisher’s exact test.

**Table 5:** Association between previous DENV/ZIKV exposure and severe dengue* during the current DENV infection.

Anti-DENV/ZIKV IgG sero-status	Severe dengue		Risk ratio (95% CI)	P value**
	No	Yes		
Prior DENV and ZIKV exposure	103	8	Not calculated	0.002
No exposure to both infections	133	0		
Prior DENV exposure only	163	16	Not calculated	< 0.001
No exposure to both infections	133	0		
Prior DENV and ZIKV exposure	103	8	0.81 (0.36–1.82)	0.667
Prior DENV exposure only	163	16		

*Define according to WHO 2009 clinical classification of dengue.

**Calculated as two-tailed p value with Fisher’s exact test.

The confounding effect of age, gender and infecting serotype for the outcome of plasma leakage was assessed with logistic regression (other outcomes were not compared due to the smaller number of observations). Both male gender (p = 0.003) and prior exposure to both DENV and ZIKV viruses (vs. no exposure to both viruses, p = 0.028) were independently associated with an increased risk of plasma leakage. Separately, younger age (p = 0.019), male gender (p < 0.0001) and previous DENV exposure alone (vs. non-exposure to both viruses, p < 0.0001) were also independently associated with plasma leakage. Serotype of current dengue infection did not influence this outcome (supplementary Table S1).

## Discussion

In this study, combination of prior ZIKV and DENV exposure was associated with a higher risk of plasma leakage and other adverse outcomes during a subsequent DENV infection in adults. However the same was true for the group with prior DENV exposure alone, and the independent effect of prior ZIKV exposure could not be discerned due to the low number of patients with prior ZIKV exposure alone. These findings also highlight the possibility of past Zika outbreaks in Sri Lanka, where acute cases have never been diagnosed. Interestingly there was no age difference between the groups with and without anti-Zika antibodies (unlike for anti-dengue IgG) suggesting that Zika might be a recent introduction to the community.

Given the structural similarities (shared epitopes of structural proteins) between ZIKV and DENV, infection with one virus is known to induce cross-reacting, but non-neutralising antibodies against the other. Therefore, it has been speculated (and supported by evidence from in vitro and animal studies) that a prior DENV infection may influence the severity of a subsequent ZIKV infection, and vice versa^2–4,7–10,25^. In effect, the ZIKV infection is thought to behave as a prior DENV infection from a different serotype leading to antibody dependent enhancement^6^. As mentioned above, clinical evidence for this hypothesis is available from a single recently published study by Katzelnick et al.^6^ which shows the risk of severe dengue to be higher on the background of a prior Zika infection for children. There are some fundamental differences between this prior study and ours which prevent a head-to-head comparison. Firstly, our study is based on an adult cohort. Adults living in a dengue endemic countries are more likely to have been exposed to multiple DENV serotypes, and with each infection the risk of a subsequent severe dengue infection may decline^6^. At the same time, it is difficult to find adult patients who had only been exposed to ZIKV, but not to DENV when the transmission of DENV has been endemic for decades. Katzelnick et al. made observations from a community-based cohort and therefore could associate prior ZIKV exposure with symptomatic DENV infection while our cohort is a hospital-based cohort which only recruited symptomatic dengue patients. Thus we could only associate prior Zika or dengue exposure with adverse dengue outcomes (not symptomatic disease). Both studies had common ground in identifying one of the adverse outcomes as “severe dengue” as defined according to WHO 2009 classification, but here also Katzelnick et al. combined both the “dengue with warning signs” and “severe dengue” categories together (hence the higher number of events) while we did not combine these two groups (hence the lower number of events). Katzelnick et al. also observed the risk of dengue haemorrhagic fever (WHO 1997 clinical classification) to be high with a prior ZIKV infection but this outcome was not observed in this study given the criticisms associated with the same classification^26^. Instead, plasma leakage which is an essential component for DHF was recorded. In this study, age and gender were iindependently associated with plasma leakage. However, these demographics may be cohort specific and cannot be generalised. For example, CDS had an over enrolment of men compared to women.

Although the present study did not diagnose acute ZIKV infection by RT-PCR, or serological (IgM or acute and convalescent IgG measurements) investigations, it provides first evidence for past ZIKV outbreaks in Sri Lanka. Globally, Zika infection was first described in 1947^27^ but came to prominence with multiple outbreaks in Pacific islands and Latin America from 2007 onwards due to concerns of the post-infection sequalae of microcephaly in infants born to infected mothers^28,29^. With increased surveillance, more cases (and outbreaks) were reported from many other countries including those in Asia. These include outbreaks in India in 2018^30^ and in Singapore in 2016^31^, two countries with a high volume of international travel with Sri Lanka. There is high potential for imported infections to have onward local transmission in Sri Lanka given the abundance of the vector mosquito (*Aedes aegypti*). Notably in 2021, an outbreak was reported in the Kerala state of India which is geographically close to Sri Lanka. In this instance 70 acute Zika cases were identified from 590 (11·9%) surveillance samples across the state collected within just 3 weeks^32^. WHO currently maintains that no Zika infections have been identified in Sri Lanka^33^, but it should be noted that acute febrile illnesses with a likely viral aetiology are not routinely tested for Zika infection and to our knowledge there is no active surveillance of at-risk people. Recently serological evidence emerged for the existence of West-Nile virus in Sri Lanka, another flavivirus unreported in Sri Lanka until specific diagnostic tests were undertaken as part of a research project^34^. Therefore, the findings reported here highlight possibility of endemic Zika infections which could be confirmed by proactive testing of symptomatic patients.

A limitation of this study is the potential cross reactivity of either anti-DENV or ZIKV antibodies across the two ELISA kits^35,36^. Given that Zika infections had not been previously reported in Sri Lanka, there may be reasonable doubt that the Zika “positive” cases are cross-reactivity from anti-dengue IgG of previous infections. However, the two ELISA kits used in this study captured antibodies against two different antigens of each virus (anti-NS1 IgG in Zika ELISA and anti-Envelope IgG in the dengue ELISA), and while the dengue ELISA kit may have high cross-reactivity with anti-Zika IgG from previous infections (false positives for dengue), the converse of false Zika positives (due to anti-dengue IgG) is less than 1% according to manufacturer’s data^21,22^. This probably explains the statistically significant difference in the quantitative anti-DENV IgG reading between people positive for IgG against both viruses vs. those only positive for anti-DENV IgG. The lack of an age difference in the two groups with and without Zika IgG antibodies also matches the epidemiological narrative of more recently introduced Zika outbreaks in contrast to long-term endemic dengue infections (where an age difference was observed, indicating exposure in childhood). Finally, only 38% of dengue IgG positive cases were also positive for Zika IgG, and in ten of these instances the semi-quantitative Zika IgG reading was higher the dengue reading. Further, in two instances patients were positive for Zika antibodies without having dengue antibodies. Given these findings it is likely that these assay results are attributable to past ZIKV exposure than cross-reactivity from dengue infections. However absolute proof of endemic Zika transmission in Sri Lanka requires diagnosis of acute cases by RT-PCR.

In conclusion, our findings show that combined prior ZIKV and DENV exposure is associated with adverse disease outcomes during a subsequent DENV infection in adults. However, the independent contribution of prior ZIKV exposure for these outcomes could not be discerned due to the low number of patients with prior ZIKV exposure alone. These results highlight the need of proactive diagnostic testing for ZIKV in at-risk symptomatic patients when clinically indicated.

## Supplementary Information

Supplementary Information.

## Data Availability

The datasets generated during and/or analysed during the current study are available from the corresponding author on reasonable request.
