# Supplementary material for: Effect of prior Zika and dengue virus exposure on the severity of a subsequent dengue infection in adults
Source: Sci Rep. 2022 Oct 14;12:17225. doi: 10.1038/s41598-022-22231-y (PMC9568574; doi:10.1038/s41598-022-22231-y)
Supplement: Supplementary file 1 — Supplementary Information. [file 41598_2022_22231_MOESM1_ESM.pdf]

Effect of prior Zika and dengue virus exposure on the severity of a subsequent dengue infection in adults

Braulio M Valencia MD[1], Ponsuge C. Sigera MSc[2], Praveen Weeratunga MD[2], Nicodemus Tedla PhD[1], Deepika Fernando PhD[2], Senaka Rajapakse MD[2], Andrew R Lloyd PhD[1], Chaturaka Rodrigo PhD[1]

Supplementary Tables

ST1. Effect of age, gender, infecting dengue serotype, prior dengue and zika exposure (vs. no exposure to both infections) on dengue-associated plasma leakage

		B	S.E.	Wald	df	Sig.	Exp(B)	95% C.I.for EXP(B)	
								Lower	Upper
Step 1[a]	Gender(1)	-.915	.311	8.630	1	.003	.401	.218	.738
	Age	-.012	.012	.929	1	.335	.988	.965	1.012
	DZvN(1)	-.678	.308	4.850	1	.028	.508	.278	.928
	Serotype1			.550	2	.760			
	Serotype1(1)	-.232	.543	.182	1	.670	.793	.274	2.299
	Serotype1(2)	.103	.379	.074	1	.786	1.109	.527	2.331
	Constant	.904	.536	2.841	1	.092	2.470		

Coding: Gender (males=0, females=1), DZvN (Prior DENV and ZIKV exposure =0, not exposed to both infections =1)

ST2. Effect of age, gender, infecting dengue serotype, prior dengue exposure (vs. no exposure) on dengue-associated plasma leakage

		B	S.E.	Wald	df	Sig.	Exp(B)	95% C.I.for EXP(B)	
								Lower	Upper
Step 1[a]	Gender(1)	-1.218	.305	16.001	1	.000	.296	.163	.537
	Age	-.028	.012	5.500	1	.019	.973	.950	.995
	Serotype1			3.655	2	.161			
	Serotype1(1)	-.046	.498	.009	1	.926	.955	.360	2.535
	Serotype1(2)	.559	.360	2.412	1	.120	1.749	.864	3.540
	DvN(1)	-1.511	.305	24.540	1	.000	.221	.121	.401
	Constant	1.912	.529	13.047	1	.000	6.765		

Coding: Gender (males=0, females=1), DvN (Prior DENV exposure =0, no prior DENV exposure =1)
